# Supplementary material for: Silk Gland Factor 1 Plays a Pivotal Role in Larval Settlement of the Fouling Mussel Mytilopsis sallei
Source: Biology (Basel). 2024 Jun 5;13(6):417. doi: 10.3390/biology13060417 (PMC11200471; doi:10.3390/biology13060417)
Supplement: Supplementary file 1 [file biology-13-00417-s001.zip › biology-2969870-supplementary.pdf]

biology

MDPI

Supplementary Materials

Silk gland factor 1 plays a pivotal role in larval settlement of the fouling mussel *Mytilopsis sallei*

Jian He [1,2], Zhixuan Wang [3], Zhiwen Wu [3], Liying Chen [3], Jianfang Huang [1,*]

[1] Institute of Oceanography, College of Geography and Oceanography, Minjiang University, Fuzhou 350108, China; hejian@mju.edu.cn
[2] Fuzhou Institute of Oceanography, Fuzhou 350108, China
[3] College of Ocean & Earth Sciences, Xiamen University, Xiamen 361102, China; zxwang@stu.xmu.edu.cn (Z.W.); wuzhiwen0717@foxmail.com (Z.W.); 22320190154004@stu.xmu.edu.cn (L.C.)
* Correspondence: jianfhuang@foxmail.com

FP1: ATGGATCTGTTTTTTTGTCATCTGGTTTATCGGGGACATATGGACCTGTTTTCTTATCATATGGCCCATCGGTGTCATATGGATCTGGTTTTTTATCATAAGGTACATCGTAGTCATA
TGGACCTGGTTTTATATCATAAGGTCCATCGTAGTCATATGGACCTGGTTTCTTATCATATGGTCCATCGTAGTCATATGGACCTGGTTTTTTATCATATGGTCCATTGTAGTAATAT
GGACCTGGATTTGTATCATATGGTCCATCGTAATCATATGGACCTGGTTTCTTATAATATGGCCCATCGGAGTCATATGGACCTGGTTTGTTATCATATGGTCCATCGTAGTCATAT
GGACCTGGTTTTTTATCCTCTGATTTATCGGGGACATATGGACCTGTTTTCTTATCATATTGCCAATCGGTGCCATATGGATTTGGTTTTTTATCATCTGGTTTAA

FP2: ATGTTGTTTTCTTTCTTACTTTTGCTTGGTTGTACCCAACTTTGCCTTGGAACTGCACCGCCTCAATATGAAGATGACGAGGATGACTACAAACCAGATACAGCGTACAAGCCGTCA
CCACCGAAATATAGACCAGTAAACCCCTGTTTAAAGAAGCCATGTAAATACAATGGACAATGTAAACCCAATGGTAGCTCCTACAAATGTATCTGCAGAGGTGGATATTATGGATA
CAATTGTAATCTAAAAAACGCCTGTAAACCAAACCCTTGTAAAAATAAAAGTAAATGTGTACCTGTTGGTAAAACTTTTAAATGTATATGTAGACTTGGAAATTTTGGTAGACTTTGC
GAAAAAAATGTATGTAGCCCTAATCCTTGTAAGAACAGAGGAAAATGCGTTCCCTGGGGAAAGACAGGATATAAATGTAAATGTGGTGGAGGATACTCAGGTCCTAGATGTGAAGT
ACACGTTTGTAAACCTAATCCATGCAAGAACAAAGGAAGATGTTATCCCGATGGCAAGACAGGATATAAATGTAAATGTGTCGGAGGATACTCAGGACCTACATGTCAAGAAAACG
CTTGTATCCCTAACCCTTGTAGCAATGGAGGGAAATGTTCAGCTGACAAATTTGGAGACTACACATGCGATTGTCGTCCTGGTTATTTTGGTCCAGAATGTGAGAAATATGTGTGC
GCCCCTAATCCATGTAGAAACAATGGAAAATGCTCCTCTGACGGCAGCGGTGGTTACAAATGTAAATGTACTGGAGGATACTCAGGTCCTACATGTAATGTACATGTTTGTAAACC
CAATCCATGTGAGAACAGAGGAAAATGTATCCCAAGAGGCAATGGTTACAAATGTAACTGTAAAGGTGGATATTCGGGCCCAACTTGTGGAGAAAATGTATGTAAACCCAATCCAT
GTCAGAACAGAGGCAGGTGTTACCCTGACAACAGTGATGATGGTTTTAAATGTAAATGTGTCGGAGGATACTCAGGTCCCACATGTGAAGATAAACCAAATCCATGTAATTCTAAG
CCATGCAAAAACGGAGGAAAATGTATTTATAATGGAAGAACTTATACCTGCAAATGTGCTTATGGATACCGTGGCAGACATTGTACTGATAAAGCATATAAACCAAATCCTTGTGCT
TCAAGGCCTTGCAAAAATGGAGGAAGGTGTACTGTTAAAGGAAACGGATATGTGTGTAAATGTACTAAAGGATACAGTGGCAAATATTGTGCTCTTAAATCACCACCATCCTATGA
CGACGAAGAGTATTAA

FP3: ATGAAGAGTCTCAGCATAGTAGTTTTGGTGGCTTTAGTCCTTATTGGATCATTCGCTGTGCAAAGTGATGCAGGCGGTAATTATTATCCAAAATATAAGTATCCAAGGGGATACAAA
GGTGGATATAACGGGTATCCCAGAGGAAATTATGGTTGGAATAAAGGCTGGAAGAAAGGTCGATGGGGACGAAAATATTATTGA

FP4: ATGAAGCGGGGATTGTTCACCATCCTCCTAATTACTGCGGTGCTAGTGGTAGTTGCAGAGTCTTACGGACGAAGATATGGAGAACCAAGCGGTTACGCTAATATTGGACACAGGA
GGTATTATGAAAGAGCTATTAGTTTCCACAGACATAGTCATGTTCACGGACATCACCTTTTACATAGACACGTTCACAGACATAGCGTTTTACATGGACATGTTCACATGCATCGCG
TTTCACACAGGATAATGCACAGACATCGCGTCCTACATGGGCATGTTCACAGACATCGCGTTCTTCATCGGCATGTTCACAGGCATCGTGTTTTACATGGGCATGTTCACAGGCAT
CGCGTTCTTCATCGGCATCTTCACAGGCATCGTGTTTTACATGGGCATGTTCACAGACATCGCGTTCTACATAACCATGTTCATAGACATAGCGTTTTACATGGACATGTTCACAGG
CATCGCGTTTTACATAGACATGTTCACAGACACAATGTATTACATGGCCATGTTCACAGGCATCGCGTTCTACATAAGCATGTCCACGACCATCGCGTTCTACATAAACATCTCCAC
AAACATCAAGTTTTACATGGCCATGTTCACAGGCATCAAGTTCTACATAAGCATGTCCACAACCATCGCGTTCTACATAAACATCTCCACAAACATCAAGTTTTACATGGACATGTT
CACACGCACCGTGTTCTACATAAACATGTTCACAAACATCGTGTTCTACATAAACATCTCCACAAACATCAAGTTTTACATGGACATATTCACACGCATCGCGTTCTACATAAACATC
TCCACAAGCATCAAGTTTTACATGGACATGTTCACACGCATCGCGTGCTACATAAGCATGTTCACAAACATCGTGTTCTTCATAAACATCTCCACAAACATCAAGTTTTACATGGAC
ATGTTCACATGCATCGTGTTCTACATAAACATGTGCACAAGCATCGTGTTTTACACAAACATGTTCACAAGCATCACGTTGTACACAAGCATGTTCACAGTCATCGCGTTCTACATA
AACATGTGCACAAACATCGCGTTGAACACCAACATGTTCACAAGCATCACGTTCTACACAGGCATGTTCACAGTCATCACGTTGTTCACAGCCATGTTCACAAACATCGAGTTGTT
CATAGCCATGTCCACAAACACAACGTTGTACACAGCCATGTTCACAGACATCAAATTCTACACCGACATGTTCACAGACATCAAGTTGTACACCGACATGTTCACAGGCATCTTATT
GCTCATAGACATATTCACAGCCACCAAGCTGCTGTTCATAGGCATGTCCACACACACGTTTTTGAGGGAAATTTCAATGATGATGGGACAGATGTCAATTTGAGAATAAGACATGG
CATCATTTACTTCGGAGGAAACACATACCGTCTAAGTGGTGGTCGAAGAAGATTTATGACTCTTTGGCAGGAATGTCTTGAATCATACGGTGACAGCGACGAATGTTTTGTGCAGT
TAGGAAACCAACATCTATTTACGGTTGTTCAAGGTCATCATAGTACTTCATTCCGTTCCGATCTGAGTAATGATTTACATCCGGACAACAACATTGAGCAGATTGCAAATGATCATG
TCAATGACATTGCGCAGTCTACAGATGGTGATATCAACGACTTTGCAGATACTCATTATAATGACGTTGCTCCAATTGCAGATGTTCATGTCGACAACATAGCGCAGACTGCAGATA
ATCATGTCAAGAACATTGCACAGACTGCACATCACCATGTCAACGATGTTGCTCAGATTGCTGATGATCATGTCAACGATATTGGTCAGACTGCATATGATCATGTCAACAATATTG
GTCAGACTGCTGATGATCATGTCAACGACATTGCGCAGACTGCAGATGATCATGTCAACGCCATTGCACAGACCGCAGATGATCATGTCAACGCTATTGCTCAGACTGCAGATGA
TCATGTCAACGATATCGGCGATACAGCAAACAGTCACATTGTACGAGTACAAGGAGTTGCTAAGAATCACCTTTATGGTATTAACAAAGCAATTGGTAAACACATCCACATTTGA

FP5: ATGTTTCTTGTAACCTTTTCTGTGGTATTTTCTAGCTTTCTTAAGATATTTGTACTTTCCAGTGCGCTTAAATTTGTAATAGTATTTCTTTCCTTTTCCGTAATACTTTCCCTTATATCC
ATGACCGTGCCAATGACTTCCAGATGGATAATTGTACGCACTGCCTGGGTAATAGCCGTCACTGTAATCATCATATCCTGA

FP6: ATGAAGTCAATCCAGATGATACTCGCAGTCTTTGTGATCACCGCTCTGTGCGGAATAGTTGAATCTGGCGGCGGTAATTACAGAGGATATTGCTCTAATAAGGGTTGTCGATCAGG
ATATATTTTTTATGACAACAGAGGATATTGTAAATATGGCTCTAGTACTTACAAGTATGATTGCGGCAGGTATGCTGGATGCTGTCTTCCGCGTAACCCATACGGCAACGTCAAATA
TTACTGCACCAAAAAAAACGCATGTCCTAAAGATTTCTATTTTTATAACAACAAAGGCTCCTATTATTATAAAAGAAATGCCTTCTACGATTGTCGTTCTTACAATGGATGTTGTCTTC
GTAGTGGTTACTAA

Figure S1. Open reading frame sequences of foot proteins from *M. sallei*.

Table S1. List of sequences of primers used for qRT-PCR

Gene name	Forward primer (5′—3′)	Reverse primer (5′—3′)	Product size (bp)
FP1	CATAAGGTCCATCGTAGT	ATGATACAAATCCAGGTC	120
FP2	GGATACCGTGGCAGACAT	TTCGTCGTCATAGGATGG	177
FP3	TGGTGGCTTTAGTCCTTA	TTCTTCCAGCCTTTATTC	148
FP4	TTATGACTCTTTGGCAGGAA	CTCAGATCGGAACGGAAT	130
FP5	ACTTTCCAGTGCGCTTAA	CAGGCAGTGCGTACAATT	114
FP6	TGATACTCGCAGTCTTTG	ATAGGAGCCTTTGTTGTT	281

Table S2. Information for top-ranked 100 targeted binding compounds of SGF1

Item	Catalog ID	Compound Name	Docking score	Mw
1	HY-17383	Levomefolate (calcium)	-11.232	457.44
2	HY-B0080	Folinic acid (calcium salt pentahydrate)	-10.728	471.42
3	HY-B0445	NAD^+	-10.011	663.43
4	HY-13667	Levoleucovorin (Calcium)	-9.938	471.42
5	HY-107780B	Cyclic-di-GMP (diammonium)	-9.671	688.40
6	HY-17379	Atorvastatin (hemicalcium salt)	-9.629	557.63
7	HY-125399	PSMA-11	-9.475	946.99
8	HY-N6006	1,3,6-Tri-O-galloyl-beta-D-glucose	-9.304	636.47
9	HY-111832	1,2,3,6-Tetragalloylglucose	-9.217	788.57
10	HY-126126	S-Adenosyl-DL-methionine	-9.110	398.44
11	Z4549268235	(R)-4-((1-(2-fluorophenyl)-2-oxopyrrolidin-3-yl)amino)-4-oxobutanoate	-9.386	293.27
12	Z4271963821	2-((1S,2S,4R)-2-hydroxy-4-(3-methoxy-4-methylbenzamido)cyclobutyl)acetate	-9.235	292.31
13	Z3698201335	3,3-dimethyl-5-oxo-5-((2-oxo-1-(3-(trifluoromethoxy)phenyl)pyrrolidin-3-yl)amino)pentanoate	-9.308	401.36
14	HY-N2031	Parishin	-9.099	996.91
15	HY-B0739	Citicoline	-8.970	488.32
16	HY-13664	Folinic acid (calcium)	-8.965	471.42
17	HY-13967B	AMG 837 (calcium hydrate)	-8.928	437.43
18	HY-119695A	Simvastatin acid (ammonium)	-8.907	435.57
19	HY-B0089	Acarbose	-8.890	645.60
20	Z4296424139	(1r,3r)-3-((3-(pyridin-4-yloxy)phenyl)carbamoyl)cyclobutanecarboxylate	-8.994	311.31
21	Z2864121525	(R)-2-(3-((R)-3-(4-methoxyphenyl)butyl)ureido)propanoate	-8.896	293.34
22	Z3681737843	(1R,2R)-1-isopropyl-2-(((S)-2-oxo-1-(pyridin-3-yl)piperidin-3-yl)carbamoyl)cyclopropanecarboxylate	-8.852	344.39
23	Z3067539094	4-((3-(methylsulfonamido)phenyl)amino)-4-oxobutanoate	-8.770	285.30
24	Z4283383471	(S)-4-hydroxy-3-((R)-3-(4-methoxyphenyl)butanamido)butanoate	-8.732	294.32
25	HY-15407A	Sacubitril hemicalcium salt	-8.753	410.48
26	HY-112889B	Oxythiamine diphosphate (ammonium)	-8.642	425.29
27	HY-N5070	Depressine	-8.609	688.63
28	Z3002591694	(1r,3r)-3-(((2-methoxynaphthalen-3-yl)methyl)amino)cyclobutanecarboxylate	-8.617	284.33
29	Z3050382907	1-(2-((5-carbamoyl-2-methylphenyl)amino)-2-oxoethyl)cyclobutanecarboxylate	-8.604	289.31
30	Z3015969575	3-(1-methyl-3-(2-((3-(trifluoromethyl)pyridin-2-yl)oxy)ethyl)ureido)propanoate	-8.587	334.27
31	Z3017785809	(1R,2R)-2-((1-(2-fluorobenzoyl)piperidin-4-yl)carbamoyl)cyclobutanecarboxylate	-8.580	347.36
32	Z3069154256	2-(3-((5-carbamoyl-2-(piperidin-1-yl)phenyl)carbamoyl)-4-methylfuran-2-yl)acetate	-8.512	384.41
33	Z4556933035	(1s,3s)-1-(5-ethyl-3-methylfuran-2-carboxamido)-3-hydroxycyclobutanecarboxylate	-8.420	266.27
34	Z3681401053	(1S,2R)-2-((4-(2-(3,3-dimethylureido)ethyl)phenyl)carbamoyl)cyclopropanecarboxylate	-8.410	318.35
35	HY-P0131A	Laminin (925-933)(TFA)	-8.561	967.06
36	HY-112038A	GSK2983559	-8.553	537.53
37	HY-107372	Uridine triphosphate	-8.483	484.14
38	HY-B0144	Pitavastatin (Calcium)	-8.459	420.45

39	HY-N4148	Purpureaside C	-8.457	786.73
40	HY-N0023	Cistanoside A	-8.439	800.75
41	HY-17504	Rosuvastatin (Calcium)	-8.346	480.53
42	HY-P0235	CDK2	-8.250	831.92
43	Z3681736600	(1S,2R)-2-((2-bromo-5-ethylphenyl)carbamoyl)cyclopropanecarboxylate	-8.348	311.15
44	Z4359087432	4-((3-fluoro-4-methoxyphenyl)amino)-4-oxobutanoate	-8.334	240.21
45	Z4500993554	(R)-4-((1-(4-chlorophenyl)ethyl)amino)-4-oxobutanoate	-8.326	254.69
46	Z4283383465	(S)-3-(4-cyclobutylbenzamido)-4-hydroxybutanoate	-8.308	276.31
47	Z2944276126	(1r,3r)-3-((2,6-difluoro-4-methylbenzyl)amino)cyclobutanecarboxylate	-8.295	254.25
48	Z4534051694	(R)-4-oxo-4-((1-(o-tolyl)ethyl)amino)butanoate	-8.290	234.27
49	Z4464310877	2-(1-(2-(((6-methyl-2-(pyrrolidin-1-yl)pyrimidin-4-yl)methyl)amino)-2-oxoethyl)cyclopropyl)acetate	-8.286	331.39
50	Z4518027314	(2R,5R)-5-((4-(2-oxopyrrolidin-1-yl)phenyl)carbamoyl)tetrahydrofuran-2-carboxylate	-8.245	317.32
51	Z3681210082	(R)-3-((3-hydroxy-5-methylphenyl)carbamoyl)-5-methylhexanoate	-8.165	278.32
52	HY-N6831	Xylohexaose	-8.168	810.70
53	HY-B0288B	Fenoprofen (Calcium hydrate)	-8.167	241.26
54	HY-118224	BPH-715	-8.138	423.38
55	HY-P2159	Dynorphin A (1-8)	-8.090	981.15
56	HY-129603	SI-109	-8.059	835.79
57	HY-W019724	2,2-Dihydroxyacetic acid	-8.058	89.03
58	HY-122308	Militarine	-8.003	726.72
59	Z4201331474	(R)-1-((4-(3-chlorophenyl)thiazol-2-yl)methyl)pyrrolidine-2-carboxylate	-8.110	321.80
60	Z2887683882	(R)-2-((3-methoxypyrrolidin-1-yl)methyl)oxazole-4-carboxylate	-8.098	225.22
61	Z3556852333	(1R,2S)-2-((3-fluoro-4-(morpholine-4-carbonyl)phenyl)carbamoyl)cyclopropanecarboxylate	-8.072	335.31
62	Z4280355188	(R)-2-(4-cyclobutylbenzamido)-4-hydroxy-4-methylpentanoate	-8.019	304.36
63	Z3685927182	4-((1-(tert-butoxycarbonyl)azetidin-3-yl)oxy)benzoate	-7.960	292.31
64	Z3301188773	(2R,4r,8R)-8-((2,4-dimethylbenzyl)amino)-5-oxaspiro[3.5]nonane-2-carboxylate	-7.959	302.39
65	Z3301188939	(2S,4s,8R)-8-((5-chloro-2-fluorobenzyl)amino)-5-oxaspiro[3.5]nonane-2-carboxylate	-7.876	326.77
66	HY-D0827	Cy2	-7.995	418.48
67	HY-N5119	Kaempferol-3-O-(2''-D-glucopyl)-D-rutinoside	-7.877	756.66
68	HY-130237	Cinnamtannin B-1	-7.841	864.76
69	HY-N0022	Isoacteoside	-7.773	624.59
70	HY-50909	Perifosine	-7.755	461.66
71	HY-N0033	Poliumoside	-7.748	770.73
72	HY-125818	Cytidine-5'-triphosphate	-7.570	483.16
73	HY-N0669	Stevioside	-7.526	804.87
74	F9994-5360	4-(thieno[3,2-b]pyridin-7-yl)benzoate	-7.445	254.28
75	HY-N1495	Maltopentaose	-7.460	828.72
76	HY-N1968	Quercetin-3-D-glucose-7-D-gentiobiosiden	-7.389	788.66
77	HY-N2124	Parishin B	-7.360	728.65
78	Z3461863498	(1S,2R)-2-((4-(pyridin-3-yl)benzyl)carbamoyl)cyclohexanecarboxylate	-7.596	337.39
79	Z3681739114	(1R,2R)-2-((3,5-dimethoxy-4-methylphenyl)carbamoyl)-1-isopropylcyclopropanecarboxylate	-7.560	320.36

80	Z2944275657	(R)-2-((4-ethoxy-3-fluorobenzyl)amino)-2-(1-methyl-1H-pyrazol-5-yl)acetate	-7.541	306.31
81	Z2941651902	1-(((4-(difluoromethoxy)-3,5-dimethylbenzyl)amino)methyl)cyclopropanecarboxylate	-7.520	298.31
82	Z3466179106	(1R,3r)-3-(((R)-1-(5,6,7,8-tetrahydronaphthalen-2-yl)ethyl)carbamoyl)cyclobutanecarboxylate	-7.336	300.37
83	Z3068030594	(2R,4R)-4-methoxy-2-methyl-1-((2-methylbenzyl)carbamoyl)pyrrolidine-2-carboxylate	-7.033	305.35
84	HY-P1226	HAEGTFTSD	-7.353	963.94
85	HY-105066	Davunetide	-7.347	824.92
86	HY-15943	6-TAMRA	-7.294	430.45
87	HY-B1426	Iodixanol	-7.292	1550.18
88	HY-N0028	Forsythiaside A	-7.283	624.59
89	HY-14739	Choline Fenofibrate	-7.277	317.74
90	HY-N4000	Digitonin	-7.264	1229.31
91	HY-101886	5-BrUTP sodium salt	-7.168	563.04
92	HY-P1505A	C3a (70-77) (TFA)	-7.137	823.94
93	HY-P0290A	GRGDSP (TFA)	-7.135	587.58
94	HY-P0154	Epsilon-V1-2	-7.113	843.96
95	F9995-1014	3'-methoxy-[1,1'-biphenyl]-4-sulfonate	-7.091	263.29
96	HY-N0244	Theaflavin-3'-gallate	-7.081	716.60
97	HY-N6719	Fumonisin B1	-7.045	721.83
98	HY-N0259	Epimedin B	-6.975	808.78
99	HY-B0617A	S-Adenosyl-L-methionine (tosylate)	-6.974	398.44
100	HY-N2594	Isoforsythiaside	-6.960	624.59

Disclaimer/Publisher's Note: The statements, opinions and data contained in all publications are solely those of the individual author(s) and contributor(s) and not of MDPI and/or the editor(s). MDPI and/or the editor(s) disclaim responsibility for any injury to people or property resulting from any ideas, methods, instructions or products referred to in the content.
